# On Fundamental Premises for Addressing "Context" and "Contextual Factors" Influencing Value Decisions in Healthcare: Comment on "Contextual Factors Influencing Cost and Quality Decisions in Health and Care: A Structured Evidence Review and Narrative Synthesis"

**DOI:** 10.15171/ijhpm.2018.62

**Published:** 2018-06-30

**Authors:** Kristine Bærøe

**Affiliations:** Department of Global Public Health and Primary Care, University of Bergen, Bergen, Norway.

**Keywords:** Healthcare Decision-Making, Value Decisions, Contextual Factors, Normative Ideals, Methodological Challenges

## Abstract

In this commentary on Williams and colleagues’ paper, I will address some essential issues related to research on contextual factors that influence value decision-making in healthcare. Based on the presumption that scientific work requires coherence in its ontological, epistemological and methodological approaches, I identify some challenges in their text and reflect on how those challenges might be addressed. I recommend that more normative work be done to make this a comprehensive area of research and suggest that the fundamental premises structuring investigations in this field be explicitly clarified.

## Introduction

In their literature review on contextual factors that influence cost and quality decisions in healthcare, Williams, Brown and Healy synthesise evidence on factors that affect allocative and technical meso-level decisions.^1^ According to the authors, these decisions are usually subjected to the requirement of ‘draw(ing) on best evidence to maximise outcomes’ (p. 2). The authors distinguish this rational and instrumentalist model of decision-making from a model that ‘open(s) up determinations to greater levels of judgement and intuitions’ (p. 9). I welcome this attention to contextual factors and to how they may influence cost and quality decision-making in healthcare. Likewise, I value the efforts the authors have made in examining the literature and their attentiveness to technical decisions; these are important priority- and limit-setting decisions, although they occur less conspicuously than allocative decisions.

The authors have underscored the limitations of their approach. However, their discussions of some crucial premises for understanding, identifying and applying the findings of contextual factors are not fully elucidated. The lack of explicit statements about the ontological, epistemological and methodological premises of this research makes it difficult for readers to fully grasp the authors’ ambitions for further research in this area. Because investigation of the mediating role of context with respect to such decisions ‘is in its infancy,’ as the authors grant, this is a good time to ask for further investigation of the foundations of this research. The following statement holds true for any research: To consolidate the scientific status of its investigation, a research area needs to establish coherence between responses to fundamental questions, namely ‘What is the nature of reality?’ (ontology), ‘What is the nature of knowledge?’ (epistemology) and ‘How do we achieve knowledge?’ (methodology).^2^ Based on this presumption, I identify challenges in the paper that it would be valuable to see discussed in further work.

## The Relation Between Normative Ideals and Descriptive Findings

The first challenge relates to how the authors perceive the relationship between identified contextual influencing factors and what should be done in light of them. Should contextualised factors or normative ideals determine the appropriateness of a decision-making process? The authors conclude that, given the various factors that are found to influence these decisions, we should not always require them to be rational in the ‘narrow’ sense mentioned above. Rather, we should meet the demands on decision-making in terms of a ‘more responsive rationality, in which multiplicity is negotiated iteratively according to changes in context, (which) is likely to be more practically useful’ (p. 11). This statement lends itself to the interpretation that influencing contextual factors are considered sufficient for justifying (for practical reasons) the relaxation of the requirement of rational decision-making.

Ontologically considered, we cannot conclude directly from descriptive evidence how the world *is* compared to how it *should* be; additional, normative arguments are needed to take that step. An approach driven by contextualised factors will need additional arguments related to an ideal of practicality (or feasibility) in order to make it possible to identify what decision-making model the diverse factors should trigger. The normative-ideal perspective differs, in that the arguments for which it calls relate to ideals about what makes the world a better, not merely a more practical, one. According to this normative-ideal perspective, decision-makers must demonstrate competency in translating normative ideals, such as political and ethical theories on delegation of powers, responsibility and accountability, into the fussy reality of the contexts in question.

The authors do suggest some guidelines for how to structure decision-making, which implies that they are not completely in line with a contextualised–factor-driven approach. They declare that ‘in relation to information, levels of resources mobilised should be roughly commensurate with the scale and likely impact of decisions’ (p. 11) and that ‘where decisions affecting costs and quality are of significant scale and scope there is a strong normative case for involving patients and citizens’ (p. 12). These claims can be seen as representing a ‘bottom-up perspective,’ ie, one essentially based on implicit appeals to common sense (with some reference to the logic of involving stakeholders) rather than to more broadly elaborated, theoretical arguments. Clearly, implementation of these normative statements will rely heavily on decision-makers’ judgement of what counts as relevant scales, scopes and impact, making it difficult to hold them accountable for these decisions. Also, without any further argumentative justification at hand, there is a high risk that their judgement may be subjected to the undue influence of others’ interests.

I conclude that, in order to support conclusions about how decision-making should be brought out when influencing, contextualized factors are identified, we need more fine-grained normative work on how to translate political and ethical theories to these fussy, real-world settings. The authors do not address this, but it seems crucial to developing comprehensive research in this area.

A related issue is worth mentioning. We can make an analytical distinction between influencing contextual factors that formally (in terms of governing) structure the decision-making process and more arbitrary factors that affect how the decision-making is carried out. This distinction is useful because it can help us to nuance the translational, normative arguments that are demanded in order to justify or reject less ideal, rational decision-making in real-world contexts. On the one side, there are governing interventions initiated by political authorities to influence conditions for pursuing idealised, rational decision-making processes. Strict governing and monitoring by means of legal regulations, for example, leave in general little space for the discretionary organisation of rational decision-making (as the authors discuss). Such contextual factors should be addressed as a matter of delegated discretion and the distribution of political power. But then, independently of such governing structures, contextual factors can also influence what is taking place *within* the decision-making process in ways that undermine the rationality of the decision-making. For example, research information may be ignored because literature is inaccessible, historical decisions may be reproduced in the absence of a culture of critical assessment and personal motivations to invest in particular service options may drive unwarranted decisions. While the first kinds of influence may be justified or combatted according to ideal political theories about the organisation of democracy, the role of professions, the internal structures of organizations and so on, the latter kinds of influences can be discussed according to ethical and moral theories stressing, for example, criteria for promoting moral equality in decision-making and ethical care for those who cannot raise their own voices. Futhermore, practical issues of infeasibility can be evaluated in relation to arguments clarifying social acceptability. Efforts are being made to clarify how value decisions in healthcare in real-world settings – acknowledged as inevitably contextualized under the influence of factors that do not promote ideal, rational decision-making – may still be turned into reasonable decisions.^3,4^

## The Nature of Context and Contextual Factors

The second challenge concerns both ontology and epistemology: What is the ontological status of the contextual factors, and how can we establish knowledge about them? But what is a ‘contextual factor’ and a ‘context’? The authors do not provide definitions of these central terms. These concepts are slippery, and that may be the reason. Still, it leaves readers to work hard at making tentative interpretations so as to grasp their project.

Analytically, the authors distinguish the contextual factors on which they focus from psychological aspects of human decision-making and underscore that psychological and contextual factors can intersect in influencing decision-making. Thus, the contextual factors in focus are related to the more objectively identifiable circumstances framing human decision-making. Still, the scope of what is covered by ‘contextual factors’ in a ‘context’ can be stretched to various extents; a context may be seen as restricted to a limited set of certain generic kinds of factors argued as particularly relevant (according to an aim), or the number of potentially relevant contextual factors comprising a context may be taken as unlimited. To see how this latter perception is possible, consider the possibility that tensions may arise between governing instruments (such as the actions promoted by economic incentives and the actions prescribed by legally fixed aims for distribution). Such tensions can occur and have an impact on decision-making in numerous and unpredictable ways.

Because the authors stress the potential of intersecting influences and interplay within and across inner and outer contexts, in addition to vindicating an ‘ecological’ approach, they seem forced to accept that there are potentially unlimited ways by which contextual factors can influence a particular setting. On the other side, they hope future work will ‘facilitate comprehensive, multivariate factor analysis across a range of decisions’ (p. 12), which seems to support the first, limited view on this pool of factors. They also call for a taxonomy of factors (eg, ‘leadership,’ ‘culture,’ and ‘resources’) ‘that can be clearly defined, measured and analysed in different settings’ to enhance ‘generality and transferability’ (p. 4). This aim to obtain ‘generality and transferability’ may indicate that ‘contexts’ are perceived as settings predetermined by certain sets of relevant, potentially influencing factors. If ‘context’ were to be understood as a composite of (in principle) an unlimited amount of potentially influencing factors and intersecting combinations thereof, the uniqueness of every context would undermine ambitions about generality and transferability in the first place. Exactly what do the authors understand a ‘context’ and ‘contextual factors’ to be?

## How to Gain Knowledge of Contextual Factors

This leads me to a third challenge, which concerns the methodology for gaining knowledge that enables us to distinguishing between the kinds of contexts that call for a rational decision-making and those that do not. The idea of establishing a taxonomy of definitions that can be ‘measured’ appears to be a positivistic approach, but also not a very flexible strategy. There are many diverse normative aims we may care to pursue by the manner in which decision-making is carried out in healthcare systems, eg, fairness, effectiveness, quality and political ideals such as democracy. Each aim can be considered to constitute a distinct ‘normative setting’ according to which conceptualisations of ‘leadership,’ ‘culture’ and ‘resources’ may differ. When circumstances are interpreted certain factors will then appear to be relevant and others not in light of these particular ‘normative settings.’ An adequate understanding of ‘context’ in this field of research might then be taken to encompass all of the factors possibly enlightened by all diverse ‘normative settings’ according to which we might care to assess these allocative and technical decisions. Restricting definitions of influencing factors in terms of what can be agreed up on and made precise enough to be ‘measured’ (thereby excluding immeasurable factors like creative leader styles and cultural self-identification), seems to narrow the scope of potentially relevant factors inappropriately. What would be the authors’ replay to this methodological challenge?

## Conclusion

With their paper, Williams and colleagues have initiated a very interesting and valuable approach to a new area of research. To address in a comprehensive manner their claim about the practical usefulness of deviating from rational decision-making, I suggest that more nuanced normative work is needed, including discussions of political theory of democracy, distribution of delegated power, ethical conduct and acceptable notions of infeasibility. This goes for both technical and allocative decisions. In future work on this topic, I also recommend that the central ontological, epistemological and methodological premises for the research be coherently scrutinized, discussed and clarified.

## Ethical issues

Not applicable.

## Competing interests

Author declares that she has no competing interests.

## Author’s contribution

KB is the single author of the paper.
